# Statistical process control applied to mechanized peanut sowing as a function of soil texture

**DOI:** 10.1371/journal.pone.0180399

**Published:** 2017-07-24

**Authors:** Cristiano Zerbato, Carlos Eduardo Angeli Furlani, Antonio Tassio Santana Ormond, Lucas Augusto da Silva Gírio, Franciele Morlin Carneiro, Rouverson Pereira da Silva, on behalf of Laboratory of Agricultural Machinery and Mechanization

**Affiliations:** Department of Agricultural Engineering, Laboratory of Agricultural Machinery and Mechanization, São Paulo State University, Jaboticabal, São Paulo, Brazil; RMIT University, AUSTRALIA

## Abstract

The successful establishment of agricultural crops depends on sowing quality, machinery performance, soil type and conditions, among other factors. This study evaluates the operational quality of mechanized peanut sowing in three soil types (sand, silt, and clay) with variable moisture contents. The experiment was conducted in three locations in the state of São Paulo, Brazil. The track-sampling scheme was used for 80 sampling locations of each soil type. Descriptive statistics and statistical process control (SPC) were used to evaluate the quality indicators of mechanized peanut sowing. The variables had normal distributions and were stable from the viewpoint of SPC. The best performance for peanut sowing density, normal spacing, and the initial seedling growing stand was found for clayey soil followed by sandy soil and then silty soil. Sandy or clayey soils displayed similar results regarding sowing depth, which was deeper than in the silty soil. Overall, the texture and the moisture of clayey soil provided the best operational performance for mechanized peanut sowing.

## Introduction

The peanut is considered one of the most important crops among the leguminous species [1]. Peanut is recommended in crop rotation programs due to its short cycle (120–140 days) and capability to be fully mechanized. In Brazil, the peanut culture is rotated with sugarcane, especially in the region of Jaboticabal, São Paulo, where productivity has recently increased. The optimal agricultural crop rotation is essential to achieving the best production. To this end, the performance of the mechanized tractor-seeder must be optimized to increase operational quality and, consequently, to obtain a higher yield [2]. The seeders play an important role in the sowing process to ensure adequate plant population [3]. Soil provides a suitable environment for plant growth, especially in the germination phase [4].

Several factors can hinder the sowing process. Some studies suggest that variability in weather or soil conditions and the mechanized systems utilized or quality agricultural operations can create issues during the sowing process [5].

Sandy soils are recommended for peanut cultivation [6] since high porosity provides better conditions for the development of roots and pods. Similarly, clayey soils with high contents of organic matter are recommended due to the formation of aggregates, which also increases its porosity [7].

However, soil production is directly linked to moisture content, and this factor is more important than the seeder used in the sowing because moisture can reduce the cohesive forces and internal friction, reducing soil strength [8].

Natural variability creates small variations in the sowing process; the quality difference is determined by the number of seeding points required for the specific process. Therefore, machinery fit for this purpose must meet the required specifications as closely as possible [9].

Quality studies are effective for improving the performance of agricultural processes and the impact of natural factors on agricultural operations [10]. Maintenance and improvement are essential to the success of any production system, especially regarding mechanized operations that are subjected to high levels of variability due to uncontrollable natural factors [11].

Some studies have used statistical process control as quality indicators. In these studies, control charts are typically used to identify specific issues resulting from process instability [11, 12, 13].

It is hypothesized that sowing is affected by soil type and moisture content. Therefore, this study evaluated the operational quality of mechanized peanut sowing in three different soil types, with variable moisture contents, using statistical process control.

## Materials and methods

The experiment was conducted during 2014 and 2015 in the peanut cultivation areas of the municipalities of Tupã (22°00'05"S and 50°33'02"W, 475 m above sea level), Dobrada (21°30'38"S and 48°28'09"W, 580 m above sea level) and Luzitânia (21°05'25" S and 48°16'00"W, 560 m above sea level), in the state of São Paulo, Brazil.

No specific permissions were needed to access the experimental sites because all activities were performed under the supervision of the farmers, and did not involve threatened or protected species.

The soils of the experimental sites were classified [14] by particle size and texture [15] as shown in Table 1 and Table 2, respectively.

**Table 1:** Soil classification and characteristics.

Experimental Site	Mapping Unit	Soil Classification and Characteristics
Tupã, SP*	PVA 10	Ultisol Eutrophic A moderate, with gently undulating relief and sandy texture
Dobrada, SP	PVA 2	Ultisol Eutrophic A moderate, with gently undulating relief and silty texture
Luzitânia, SP	LV 45	Oxisol A moderate, with plan and gently undulating relief and clayey texture

*SP: State of São Paulo, Brazil.

**Table 2:** Grain size analysis of the soil from the experimental area.

	Clay	Silt	Fine Sand	Coarse Sand	Textural Class
Experimental Site	..........................g kg^-1^.........................				
Tupã, SP*	76	35	561	328	Sandy
Dobrada, SP	153	43	348	456	Silty
Luzitânia, SP	497	126	222	155	Clayey

*SP: State of São Paulo, Brazil.

Conventional tilling down to approximately 0.20 m was implemented, with one plowing and two harrowing passes. Ten soil samples were collected using a Dutch auger 0 to 0.20 m from each experimental site and packed in aluminum containers to determine the moisture content by the standard gravimetric method [16]. The average moisture content of the soils was 10.65%, 13,25% and 24.65% for sandy, silty and clayey soils, respectively.

**Fig 1:**
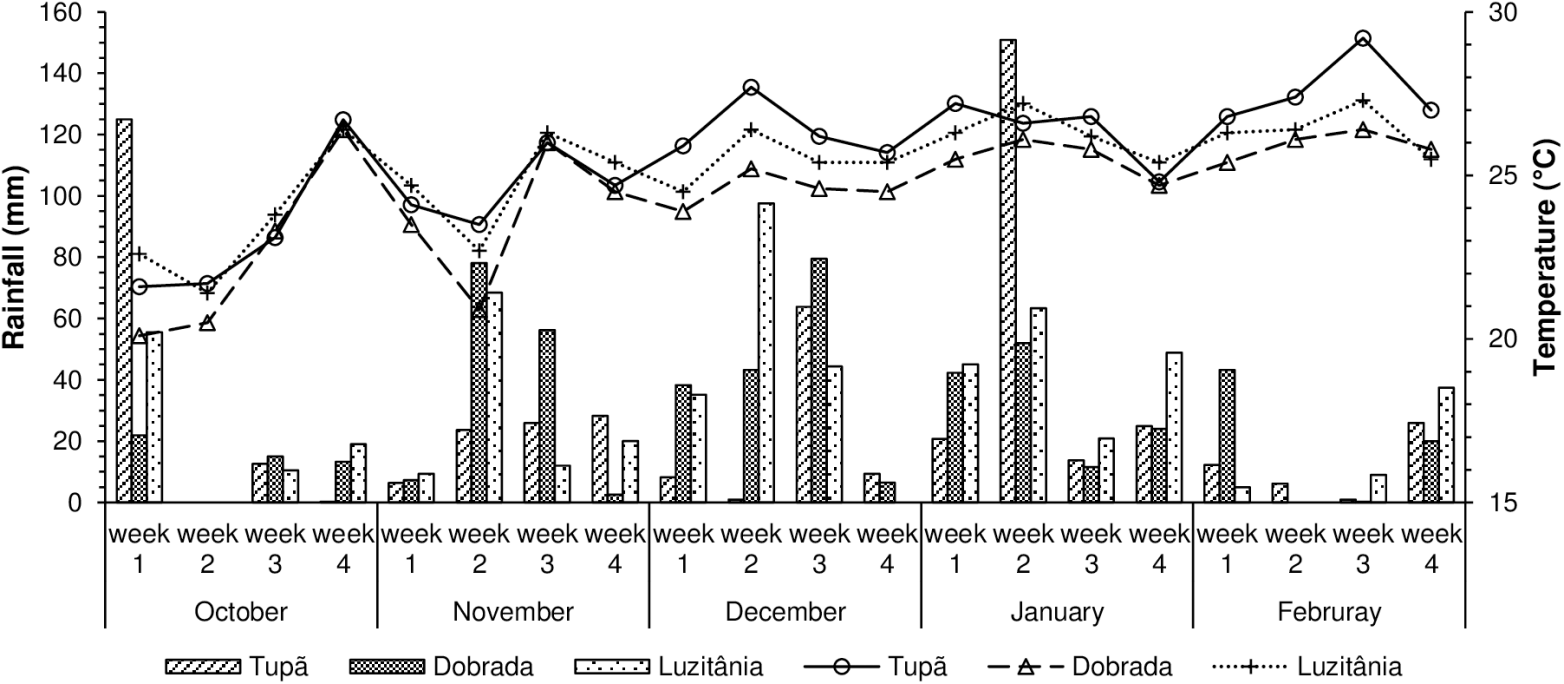
Rainfall (mm) and average temperature (°C) during the period of the experiment. Bar for rainfall and line for temperature.

Fig 1 shows the average rainfall and weekly temperature during the experimental period [17].

The track-sampling scheme was adopted for homogeneity of the fields, and 80 samples for each soil type were analyzed; samples were collected 20 at a time from 4 seeder-tractor passes, with 2 passes in the travel direction and 2 in the opposite direction for randomization. Each sample was spaced 50 m apart, parallel to each other. Treatments were determined according to the soil type in each area, which was classified as sandy, silty and clayey.

The treated peanut seeds (Arachis hypogaea L., passed through a 23 mm sieve), cultivar Granoleico, Virginia group, are characterized as a spreading type with indeterminate growth habit. The seeds had 70% germination, confirmed by the seed supplier. Another 5% was lost after passing through the seed metering mechanism in the Seed Analysis Laboratory of FCAV, UNESP, in Jaboticabal, State of São Paulo, Brazil. Therefore, the seeds had 65% germination at the time of sowing.

The following machines and settings were used for the mechanized peanut sowing:

• Sandy soil: John Deere tractor, model 6415, 4WD, with 78 kW (106 hp) of power on the engine at rated speed, operating in 2^nd^ gear C with 1600 rpm on the engine. BIA-Baldan mechanical seeder-fertilizer, 4 line (double), 0.18 x 0.77 m spacing between lines (double-spaced), equipped with dual mismatched discs to deposit the seeds and open the groove to apply fertilizer, and double press wheels in a "V."

• Silty and Clayey soils: Massey Ferguson tractor, model 7150, 4WD, with 110 kW (150 hp) on the engine at rated speed, operating in 2^nd^ gear with the engine at 2000 rpm. PHT3 Supreme Pneumatic Seeder, regulated to 4 sowing rows spaced 0.90 m with dual mismatched discs to deposit the seeds and open the groove to apply fertilizer, and double press wheels in a "V."

To create the same plant population, the settings were identical for all seeders used. The sowing depth was adjusted to 0.06 m and the operation speed to approximately 6.3 km h^-1^ because small oscillations occurred during the operation. The sowing density was adjusted to 20 seeds m^-1^. However, in the sandy soil the density was adjusted to 10 seeds m^-1^ per row because the spacing was double.

No fertilization was carried out during sowing. The sandy soil was previously planted with corn, and the silty and clayey soils were previously planted with sugarcane. Corn and sugarcane are crops that are heavily fertilized and leave residual fertilizer in the soil.

The variables used as indicators of the quality of mechanized sowing were as follows:

- Sowing density: after passing the tractor-seeder, the seeding furrow was dug for 2 m at each sampling location and the number of seeds deposited by the seeder was counted. This variable was transformed into seeds m^-1^.
- Normal seedling spacing: after seedling emergence stabilized, the spacing between them was measured using a measuring tape, 2 m from the sowing line at each sampling location. The data was analyzed to determine the spacing corresponding to a normal class based on the reference spacing (Xref) of the seeder regulation (0.5. Xref <Xi <1.5. Xref) [18].
- Sowing depth: after emergence, three seedlings were collected from each sampling location and the distance from the seed to the soil surface was measured using a graduated ruler. The final value was the average of the three measurements.
- Initial seedling stand: the number of seedlings per 2 m sowing row was determined, after emergency stabilization at each sampling location, and the value was converted to plants per hectare.

Pod productivity was determined before harvesting, by digging up all the peanut plants inside a 2 m^2^ area, collecting the pods that remained on and under the ground to approximately 0.15 m deep. The procedure was repeated in six sampling locations for each soil type. After harvesting and sieving, the pods were placed in paper bags for weighing. The moisture content of all samples was adjusted to 8%, which is the moisture for peanut storage; these values were then extrapolated to kg ha^-1^.

The data was evaluated with descriptive statistical analysis by calculating a measure of central tendency (average) and two measures of dispersion (standard deviation and coefficient of variation).

The results were also evaluated by statistical process control, using the control cards of the I-MR (individual values and moving range) type, which have center lines (general average and average amplitude). Additionally, the upper and lower statistical control limits (UCL and LCL) were calculated based on the standard deviation of the variables (for UCL, average plus three times the standard deviation, and for LCL, average less three times the deviation, when larger than zero).

Upper and lower specific control limits (USCL and LSCL, respectively) on the chart of individual values were stipulated, from the acceptable limits for each variable. A specific limit of variation in the moving range (MR) charts was determined from the difference between the specific limits, which is the largest possible variation to be accepted for the operation to be considered of good quality. Table 3 shows the specific limits for each variable.

**Table 3:** Specific control limits for each variable.

Indicators*	USCL	LSCL	MR
Sowing density (seeds m^-1^)	22	18	4
Normal spacing (%) calculated according to [23]	55	35	20
Sowing depth (cm)	8	5	3
Initial seedling stand (plants ha^-1^)	160000	130000	30000

* Sowing density: calculated at approximately 2 m^-1^. Normal spacing: calculated at approximately 10% and corrected to 65% (seed germination). Sowing depth: calculated at the regulated level of 3 cm, following previous work [6]. Initial seedling stand: calculated at 10% with respect to a theoretical population already corrected to 65% (seed germination).

These charts were used to identify the quality of the operation, using the four variables previously described as quality indicators.

## Results and discussion

All soil types were stable from the viewpoint of statistical process control; that is, the values remained between the upper and lower control limits, even with natural process variability.

**Fig 2:**
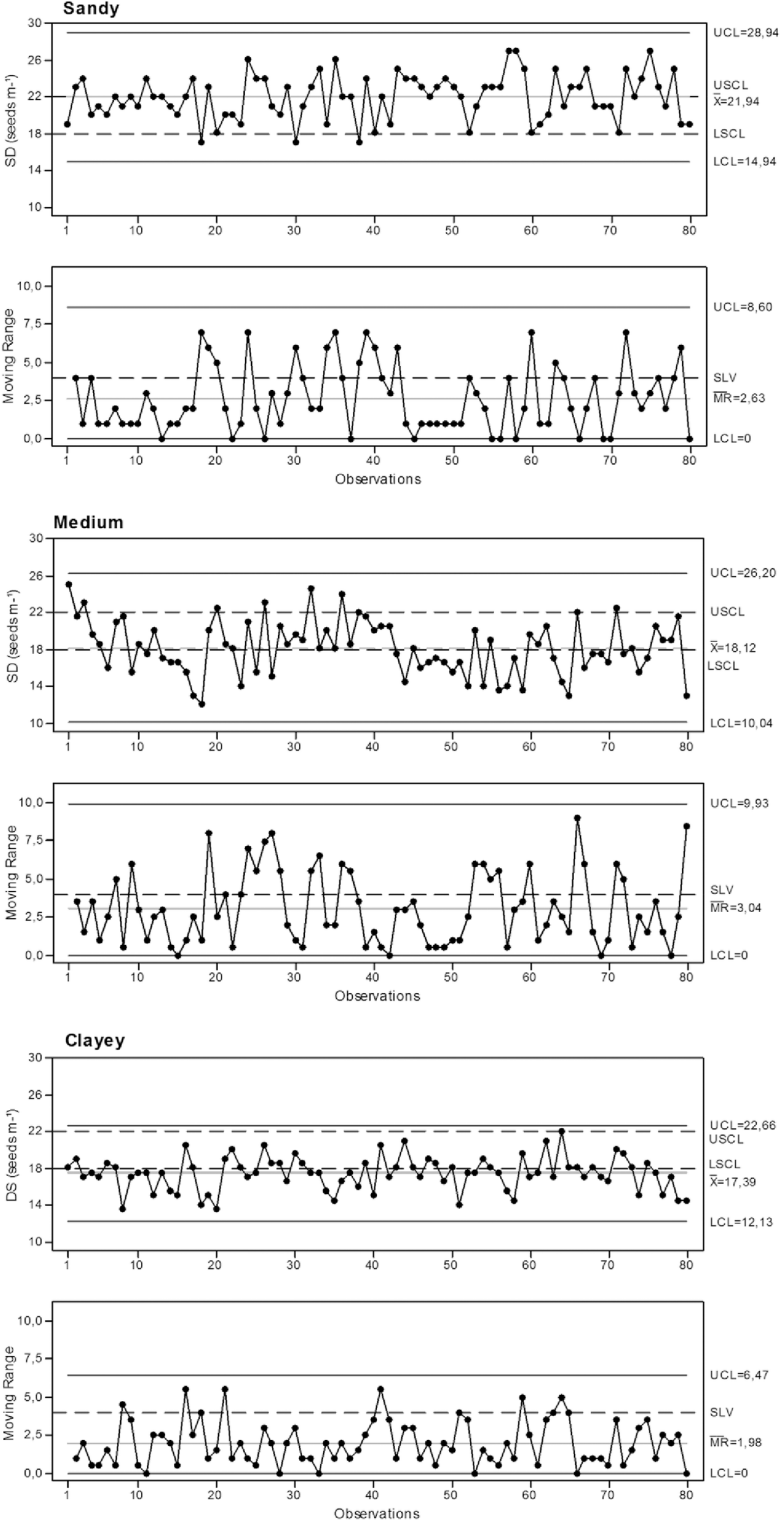
Control charts for sowing density. SD: Sowing density. UCL: Upper control limit. LCL: Lower control limit. USCL: upper specific.

For the sowing density, the silty soil had the greatest variability within the control limits (Fig 2) and had an elevated coefficient of variation and standard deviation (Table 4). For the sowing density, the silty soil type had the lowest quality among the three soil types evaluated.

**Table 4:** Percentage of samples within the specified limits for sowing density.

Soil	Control (%)			Variation (%)		CV	SD
	Acceptable	Above	Below	Acceptable	Above	(%)	(seeds m^-1^)
Sandy	52,5	43,8	3,7	81,3	18,7	11,2	2,4
Silty	43,7	8,8	47,5	72,5	27,5	16,3	3,0
Clayey	42,5	0,0	57,5	92,5	7,5	10,5	1,8

CV: coefficient of variation. SD: standard deviation.

Although the sandy soil had the highest number of samples within the specified control limits, the average was very close to the upper limit, and a large number of samples exceeded the limit (Table 4). This result indicates an excessive seed spending due to the adjustment of the metering system of the mechanical seeder-fertilizer. Clayey soil had an average sowing density very close to the lower specific limit, and no samples exceeded the USCL (Table 4). This soil type was considered the best quality due to lower coefficients of variation and standard deviation, the smallest range between the USCL and LSCL, and the most samples inside the given specification limits of variation, (LSV), even though many samples were below the lower specific control limit. The correct adjustment of the seed-metering machine and its performance during sowing improve productivity.

Continuous monitoring of the operation allows for detection of possible failures in the sowing process, which can then be corrected and maintained within acceptable quality standards. The use of statistical process control for monitoring and, consequently, developing an improvement plan to increase the quality of the operation are essential tools for companies to be able to reduce production costs due to faulty operations[19]. In mechanical sowing, seed consumption impacts production costs, and if excess consumption can be eliminated, as in the case of sandy soil, the operations become more profitable.

**Fig 3:**
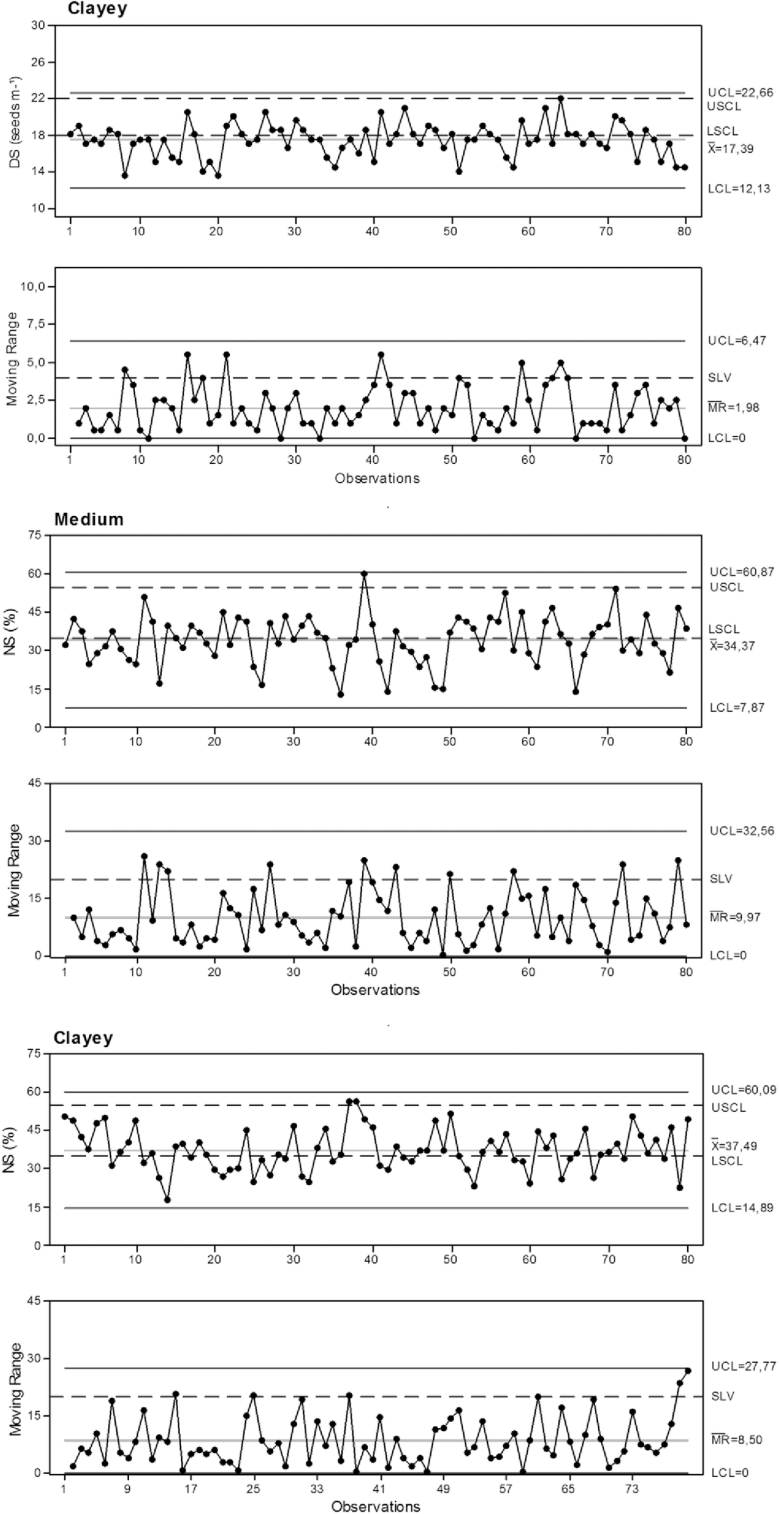
Control charts for normal spacing. NS: Normal spacings. UCL: Upper control limit. LCL: Lower control limit. USCL: upper specific.

The silty soil was classified as lower quality with respect to the normal spacing variable because fewer samples were within the acceptable limits, with most of them below the lower specified limit (Fig 3). It is noteworthy that this variable is measured between the emerged seedlings and that environmental conditions can directly influence seedling emergence. The emergence of peanut seedlings can be aided or impaired due to changes in the environment where they are sown [20].

Soil moisture content at the time of sowing was low (13.25%) and rainfall during the first month of the experiment was the lowest of all months recorded (89.8 mm). For this reason, seedling emergence may have been lower than average, decreasing the quality of mechanized sowing. It is also emphasized that the sowing density for this soil was the lowest, which increased normal seedling spacing.

Although sandy soil had the most samples within the acceptable standard for normal seedling spacing (Table 5), it also had many samples lower than the specified limit, in addition to the highest variability.

**Table 5:** Percentage of samples within the specified limits for normal spacing.

Soil	Control (%)			Variation (%)		VC	SD
	Acceptable	Above	Below	Acceptable	Above	(%)	(%)
Sandy	60,0	8,8	31,2	83,5	16,5	29,2	11,7
Silty	47,5	1,3	51,5	87,3	12,7	27,7	9,5
Clayey	58,7	2,5	38,8	92,4	7,6	22,0	8,3

VC: variation coefficient. SD: standard deviation.

The clayey and sandy soils displayed similar results for normal seedling spacing. The clayey soil had the most samples within the acceptable specified limits (individual chart values), and a certain number of samples below the specified lower limit. However, unlike the sandy soil, it had the smallest variability and almost all samples were within the accepted variation limit (moving range chart), classifying this soil as higher quality for normal seedling spacing.

The difference in the qualitative performance of the soils (sandy and clayey) resulted from the soil types and conditions at sowing. With adequate fertilization, light textured (sandy) and well-drained soils are preferred for peanut cultivation, but soils with up to 20% clay are also acceptable [6]. This study showed that even with low moisture content (10.65%), sandy soil produced good emergence of seedlings after sowing. Seedling emergence is more difficult in clayey soils due to its more compact structure. However, with correct soil preparation and appropriate moisture content for sowing (24.65%), seedling emergence in clayey and sandy soils was similar and enhanced performance with respect to normal spacing. Furthermore, rainfall in the areas with clayey and sandy soils was similar during the experiment (Fig 1).

**Fig 4:**
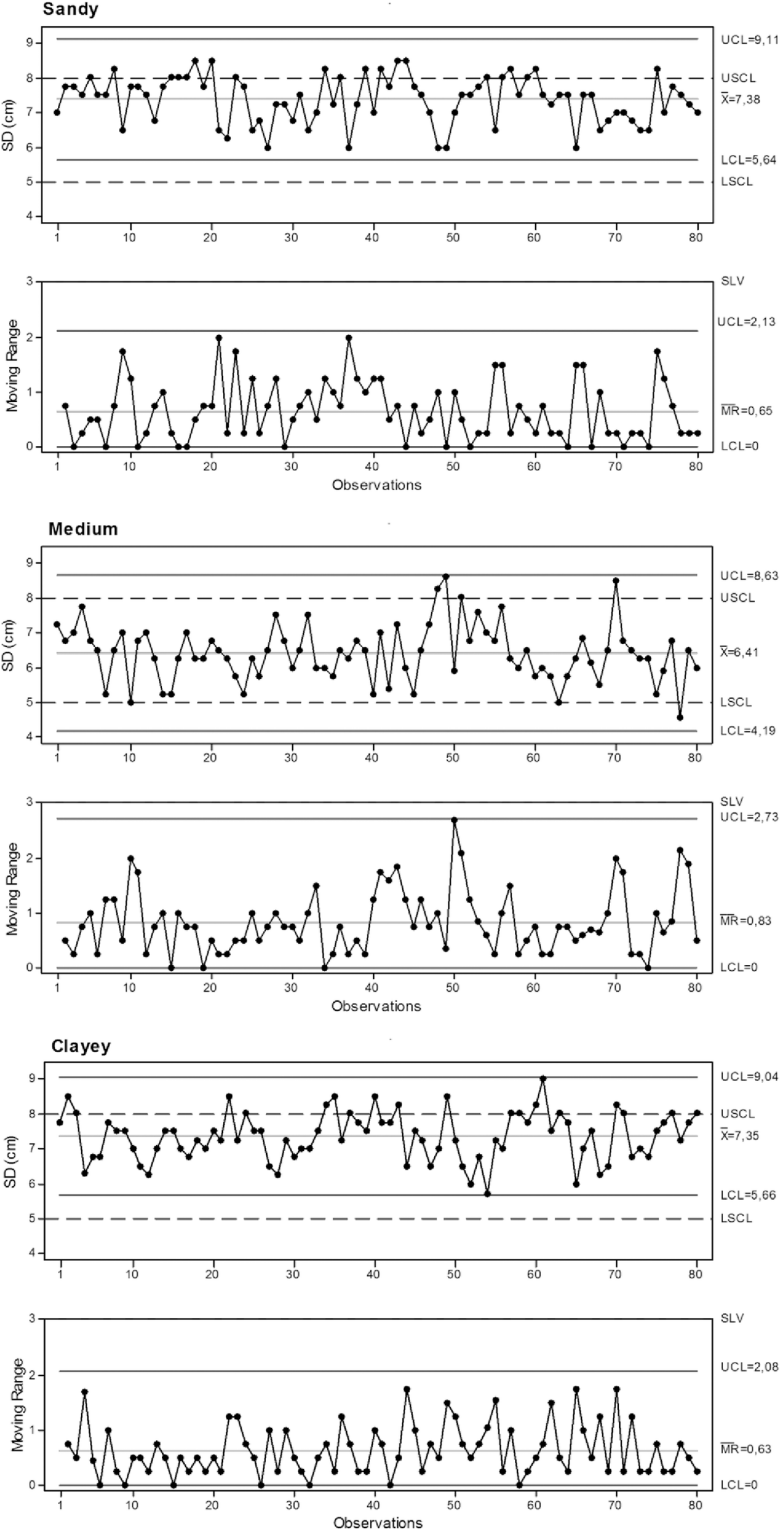
Control charts for sowing depth. SD: sowing depth. UCL: Upper control limit. LCL: Lower control limit. USCL: upper specific.

The sowing depth in sandy and clayey soils had few samples above the upper specific limit (Fig 4), indicating deeper sowing than desirable at these locations. However, quality standards were maintained because all the samples were within the specified limit of variation (moving range chart).

The average sowing depth for silty soil was within the specified control limits and within the specified limit of variation for nearly all samples (Table 6), which classified this soil as the best quality for sowing depth.

**Table 6:** Percentage of samples within the specified limits for sowing depth.

Soil	Control (%)			Variation (%)		VC	SD
	Acceptable	Above	Below	Acceptable	Above	(%)	(cm)
Sandy	86,2	13,8	0,0	100,0	0,0	9,1	0,67
Silty	96,2	3,8	1,3	100,0	0,0	12,6	0,81
Clayey	87,5	12,5	0,0	100,0	0,0	9,4	0,69

VC: variation coefficient. SD: standard deviation.

The sowing depth is an important quality indicator because inadequate sowing depths can affect how the seeds develop, resulting in weaker growth performance [21]. The recommended sowing depth for peanuts is 5 to 8 cm [6], and there is no difference in peanut production for sowing depths of 6 and 8 cm [1]. In this study, no soil type had an average sowing depth deeper the recommended 8 cm, maintaining the quality standard.

**Fig 5:**
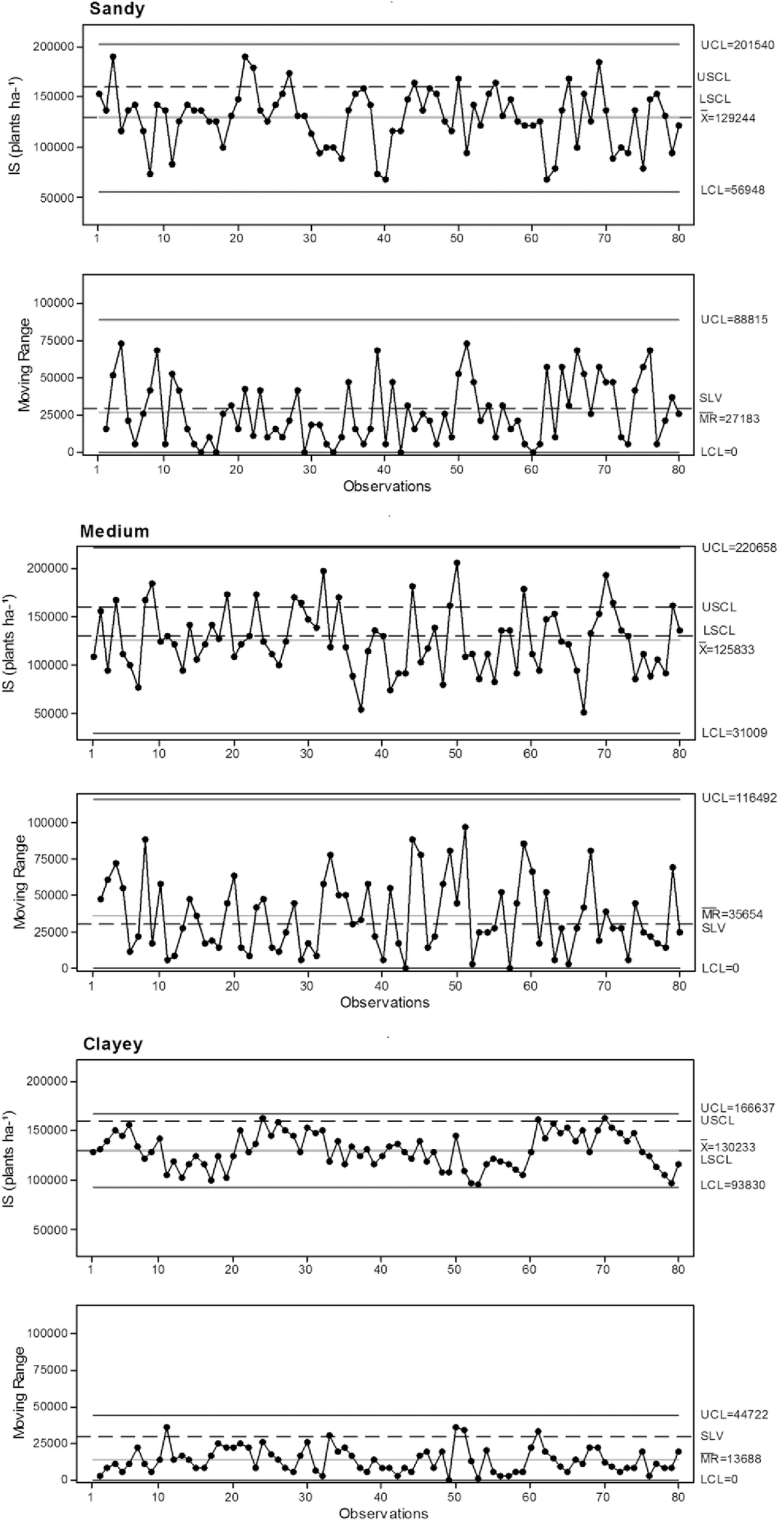
Control charts for initial seedling stand. IS: initial seedling stand. UCL: Upper control limit. LCL: Lower control limit. USCL upper specific.

Silty soil displayed low-quality initial seedling stands, with a large number of samples below the specified lower limit and above the variation limit (Table 7), a high coefficient of variation and standard deviation, and a wide range between the upper and lower limits of the statistical control (Fig 5). The sowing depth and normal spacing parameters negatively influenced the results of the initial seedling stand.

**Table 7:** Percentage of samples within the specified limits for the initial seedling stand.

Soil	Control (%)			Variation (%)		VC	SD
	Acceptable	Above	Below	Acceptable	Above	(%)	(plants ha^-1^)
Sandy	43,7	11,3	45,0	60,8	39,9	21,8	28217
Silty	23,7	20,0	56,3	53,2	46,8	26,1	32836
Clayey	42,5	3,8	53,7	93,7	6,3	13,2	17227

VC: variation coefficient. SD: standard deviation.

Sandy or clayey soils behaved similarly regarding the number of samples within the specified acceptable limits. However, they differed with respect to process variation, where clayey soil was clearly better. Clay displayed less variability since almost all samples were within the acceptable variance limits, had lower coefficients of variance and standard deviation, and had the smallest range between the upper and lower limits of statistical control.

In synthesis, higher quality sowing density and normal spacing gave the clayey soil a more homogeneous initial seedling stand, as expected. A population density of 130,000 plants ha^-1^ can achieve great productivity [6]. The sandy soil had the greatest average sowing density, and it resembled the clayey soil regarding the number of samples within the specified limits for the other variables. However, moisture conditions during sowing affected its quality by inducing greater variability. Silty soil, with the exception of density and sowing depth, as determined by machine performance, was of lower quality compared to other soil types due to low moisture content and rainfall.

Numerically, pod productivity was 5,497 kg ha^-1^, 5,299 kg ha^-1^, and 5,089 kg ha^-1^ for clayey, sandy and silty soils, respectively. The differences, on average, of 198 kg ha^-1^ (3.6%) between clayey and sandy, 210 kg ha^-1^ (4.0%) between sandy and silty, and 408 kg ha^-1^ (7.4%) between clayey and silty are largely a consequence of the evaluated quality of the indicators discussed previously since productivity results from several biotic and abiotic factors during the crop cycle. Furthermore, the productivities reached in this study were good considering that the national and São Paulo state averages are 3,007 and 3,131 kg ha^-1^, respectively [22].

## Conclusion

For the three textural classes of soil, all variables remained within the control limits.

Clayey soil had higher quality sowing density, normal spacing and initial seedling stand, followed by sandy and silty soils.

For the sowing depth variable, silty soil performed better, followed by sandy and clayey soils, which behaved similarly for this variable.

Overall, the results showed that although the clayey soil had clay content above what is recommended for peanut crops, it performed better due to more favorable moisture conditions.

## Supporting information

S1 File Supporting results. Data Thesis. https://doi.org/10.6084/m9.figshare.4204665.v1.
(RAR)
